# Evolving the Scope of Cardiac Point-of-Care Ultrasound in the Current Era

**DOI:** 10.7759/cureus.53985

**Published:** 2024-02-10

**Authors:** Sameer Maheshwari, Himansu Dagor

**Affiliations:** 1 Medicine, Sri Aurobindo Institute of Medical Sciences, Indore, IND

**Keywords:** real-time ultrasonography, miniaturization of technology, cardiac care, internal medicine (general medicine), point-of-care ultrasound (pocus)

## Abstract

Point-of-care ultrasound (POCUS) has become a flexible and multifaceted diagnostic instrument in the realm of cardiac care, transforming the landscape of cardiovascular assessment. This review aims to explore the extensive scope of POCUS applications in cardiac care, highlighting its diverse utility across various medical specialties. POCUS, conducted at the patient's bedside, offers real-time insights into cardiac anatomy and function, providing a valuable adjunct to traditional diagnostic methods. In critically ill patients, POCUS has demonstrated its effectiveness in the rapid evaluation of the left and right ventricular function, identification of pericardial effusion and tamponade, assessment of volume status, and detection of valvular lesions. Its role as an adjunct to the physical examination has been particularly impactful, leading to early diagnoses and significantly influencing medical management decisions. The review also discusses the current limitations of POCUS technology. As the utilization of POCUS continues to expand across diverse medical disciplines, its ability to offer timely and accurate diagnostic information is poised to reshape the standard of care in cardiac medicine. This comprehensive review provides insights into the evolving role of POCUS in cardiac care and underscores its potential to enhance patient outcomes through rapid and informed decision-making at the point of care.

## Introduction and background

In recent decades, there has been a growing interest in the miniaturization of technology, extending to advancements in ultrasound technology [1]. One notable application is in cardiac ultrasound, or echocardiography, which serves as a noninvasive diagnostic tool for cardiac patients [2]. As the primary method for diagnosing heart conditions, echocardiography utilizes ultrasound to examine the heart [3]. The landscape of cardiovascular ultrasound has shifted over time, moving from dedicated echocardiography labs to the hands of nontraditional users [4].

Currently, healthcare professionals have embraced point-of-care ultrasound (POCUS), a practice where providers can conduct real-time ultrasonography at the patient's bedside [1]. POCUS involves the use of handheld ultrasound machines that are more portable compared to traditional full-platform systems. This approach allows healthcare providers to directly correlate ultrasound findings with the patient's presenting signs and symptoms. Furthermore, the evaluation can be repeated as needed based on any changes in the patient's condition. This evolution in ultrasound technology and its expanded accessibility contribute to more dynamic and responsive patient care [5].

## Review

Cardiac POCUS and current usage of the technology across specialties

POCUS for cardiac diagnosis involves the assessment of the heart and its vessels by a healthcare practitioner directly at the patient's bedside using ultrasound [4]. Various machines are employed for these examinations, spanning from compact handheld, battery-powered devices to portable ultrasound machines. Advancements in technology have given rise to the development of application-based ultrasonography, allowing users to transform their smartphones or tablets into portable ultrasound devices by connecting to a transducer. Furthermore, certain contemporary devices feature touch screens for enhanced usability [6].

The characteristics of cardiac POCUS may differ based on the specialty, but a shared attribute across these specialties is its differentiation from the standard echocardiogram [4]. A detailed comparison between POCUS and the comprehensive full echocardiogram is outlined in Table 1.

**Table 1:** Difference between POCUS and standard full echocardiogram PCOUS: point-of-care ultrasound, 3D echo: three-dimensional echocardiography Citation: Lee and DeCara [1]

Parameter	POCUS	Echocardiogram
General attributes [1]		
Location	Bedside, often in a clinical setting	Typically conducted in a specialized echocardiography lab or imaging facility
Purpose	Rapid assessment, immediate diagnosis	Comprehensive evaluation, detailed assessment of cardiac structure and function
Handler	Healthcare practitioner	Sonographer
Protocol	Limited, can be part of the multiorgan evaluation	Complete evaluation
Availability of results	Real time	Some delay
Training requirement	Brief, variable	Extensive and standardized
Cost	Generally lower cost due to simplified equipment	Often higher cost associated with specialized equipment and dedicated facilities
Use Cases	Immediate bedside diagnosis, monitoring changes in real-time	Detailed assessment for complex cardiac conditions, pre-surgical planning, and follow-up evaluations
Machine attributes [1]		
Size	Ultra-portable, lightweight	Large, bulky, occupy more space
Image quality	Adequate for many applications, however, there can be limitations in obese and vented patients	Highest resolution
Color flow Doppler	Available	Standard
Spectral Doppler	Limited availability	Standard
3D Echo	Not available	Available
Artificial Intelligence	In development	In development

POCUS offers inherent flexibility compared to the more formal requirements of a standard echocardiogram. The extent of this flexibility varies depending on factors, such as available resources, institutional guidelines (spanning from care delivered at home to clinics and encompassing every facet of the hospital), and established policies. The degree of freedom also evolves in accordance with the scope of practice among different specialties [4]. Cardiac POCUS is widely utilized across various medical specialties, indicating its extensive applicability. The adaptability of POCUS to different settings and specialties underscores its versatility as a diagnostic tool, allowing for more dynamic and immediate assessments in diverse healthcare environments.

Scope of Cardiac POCUS in Anesthesiology

Anesthesiologists employ cardiac POCUS for a comprehensive range of evaluations, including the assessment of volume status, hemodynamics, cardiomyopathy, myocardial ischemia, valvular abnormalities, pericardial conditions, ventricular abnormalities, identifying the capture of pacing signals, and during cardiac arrest situations [4,7,8]. This broad spectrum of applications highlights the utility of cardiac POCUS in aiding anesthesiologists in various aspects of patient care. Furthermore, several reputable organizations, such as the American Society of Regional Anesthesia (ASRA), Canadian Anesthesiologists’ Society (CAS), and Society of Critical Care Anesthesiologists (SOCCA), have recommended and endorsed the use of cardiac POCUS [4,8,9]. These endorsements underscore the recognized value and importance of incorporating cardiac POCUS into the practice of anesthesiology, emphasizing its role in enhancing diagnostic capabilities and decision-making in perioperative and critical care settings.

Scope of Cardiac POCUS in Cardiology

The American Society of Echocardiography (ASE) advocates for the use of POCUS in the field of cardiology [4,10,11]. This recommendation underscores the significance of incorporating POCUS into cardiology practice. The core cardiac applications of POCUS within the cardiology specialty encompass a range of evaluations, including size and function of the ventricles, assessing increased pressures during left heart filling, detecting abnormalities in heart valves, evaluation of enlargement of the left ventricle, diagnosis of hypertrophic cardiomyopathy, identification of aortic aneurysms, detection of pericardial effusion, and utilization during cardiac arrest situations [4]. These key applications highlight the versatility and diagnostic value of cardiac POCUS in addressing various aspects of cardiac health and pathology within the field of cardiology. The ASE's endorsement emphasizes the importance of integrating POCUS as a valuable tool for cardiologists in their clinical assessments and decision-making processes.

Scope of Cardiac POCUS in Critical Care

POCUS finds extensive application in the field of critical care, where it is utilized to assess various aspects of cardiac health. In critical care, POCUS is employed for the evaluation of shock, size and function of ventricles, identification of irregularities in the motion of the heart wall, and detection of irregularities in the heart valves [4]. Notably, basic cardiac POCUS has been incorporated into the educational programs for critical care medicine in Australia and Europe, emphasizing its fundamental role in the education and practice of critical care professionals [4,12,13]. In India and Canada, guidelines acknowledge and recommend the use of POCUS in critical care settings, further establishing its importance as a valuable diagnostic and management tool in the context of critically ill patients [14,15].

Scope of Cardiac POCUS in Emergency Medicine

Emergency medicine physicians were the pioneers among various specialists in adopting limited POCUS, initially focusing on identifying cardiac tamponade or significant reductions in left ventricular (LV) function. In the emergency medicine setting, cardiac POCUS serves several purposes, including the evaluation of the ventricular size and function, identification of pericardial effusion, assessment of volume status, providing direction for pericardiocentesis and verifying the placement of transvenous pacing wires, and detection of aortic aneurysms [16,17]. This application of cardiac POCUS in emergency medicine contributes to a more focused differential diagnosis for individual patient encounters, guiding subsequent care and providing essential support in the diagnosis of emergent conditions. Recognizing its value, both the American College of Emergency Physicians (ACEP) and the Canadian Association of Emergency Physicians (CAEP) endorse and recommend the use of cardiac POCUS in emergency settings [16,17]. This endorsement highlights the significance of integrating POCUS into emergency medicine practice for timely and effective patient care.

Scope of Cardiac POCUS in Internal Medicine

Cardiac POCUS has become widely prevalent and is experiencing rapid growth in its use within the field of internal medicine. Among the early adopters of cardiac POCUS were internal medicine specialists in critical care, inspired by the practice's success in emergency medicine for promptly assessing patients in acute distress. However, in the past decade, the adoption of POCUS has expanded significantly across various domains within internal medicine, with hospitalists, primary care providers, and numerous subspecialists incorporating it into their diagnostic and monitoring practices.

Numerous prominent professional organizations in internal medicine, such as the Academic Alliance for Internal Medicine, the American College of Physicians, the Society of Hospital Medicine, and the Society of Critical Care Medicine, now officially support the use of POCUS, including its applications in cardiac assessment. This endorsement reflects the growing acknowledgment and acceptance of POCUS as a valuable tool within the realm of internal medicine [4,15,18].

While there has been a notable rise in the utilization of cardiac POCUS in internal medicine and several major organizations endorse its use, the National Medical Council of India has incorporated formal training in their postgraduate medicine curriculum. Nevertheless, the POCUS training landscape is evolving swiftly globally. At present, there are emerging opportunities for advanced POCUS training through internal medicine POCUS fellowships post-residency.

The core cardiac applications of POCUS in internal medicine encompass the assessment of ventricular size and function, identification of pericardial effusion, evaluation of atrial size, examination of valvular function, determination of volume status, and detection of aortic aneurysms [4]. These applications demonstrate the versatility of cardiac POCUS as a valuable diagnostic tool in internal medicine, aiding clinicians in the comprehensive evaluation of cardiac health for more informed decision-making.

Scope of Cardiac POCUS in Obstetrics-Gynecology

Cardiac POCUS plays a crucial role in evaluating fetal cardiac activity at the bedside and using Doppler assessment to examine fetal circulation within the field of obstetrics-gynecology. Recognizing its significance, the Society of Obstetricians and Gynecologists of Canada recommends the use of cardiac POCUS in this setting [4,19]. This endorsement underscores the valuable contribution of cardiac POCUS in enhancing the diagnostic capabilities of healthcare professionals in obstetrics-gynecology, particularly in the evaluation of fetal cardiac health and circulation.

Assessment of cardiac function using POCUS

Cardiac POCUS is not equivalent to an echocardiographic evaluation. In usual settings, POCUS is often complemented by a comprehensive transthoracic echocardiogram (TTE) to validate POCUS findings and identify additional abnormalities that may not be easily discerned through POCUS alone [6]. The POCUS will help in the evaluation of the function of the left ventricle, valvular disease, functions of the right ventricle, pericardial conditions, and volume status. Cardiac POCUS should not be considered a direct substitute for a comprehensive echocardiographic evaluation. Typically, in routine clinical settings, POCUS is often followed by a complete TTE to validate POCUS findings and to identify any additional abnormalities that may not be easily discernible through POCUS alone [6]. POCUS serves as a valuable tool in the initial assessment, providing insights into various aspects of cardiac health, including LV function, valvular disease, right ventricular (RV) function, pericardial disease, and volume status. However, its role is primarily as a rapid and focused assessment, and the follow-up with a complete TTE ensures a more comprehensive and detailed evaluation of cardiac structure and function [6]. The combination of POCUS and subsequent TTE allows for a more thorough understanding of cardiac conditions, enabling healthcare professionals to make well-informed diagnostic and treatment decisions.

Assessment of LV Function

In individuals exhibiting minimal physical examination findings suggestive of LV systolic dysfunction, POCUS proves valuable by offering an assessment of ejection fraction and detecting increased pressures during LV filling. This is particularly relevant in critically ill patients within the intensive care unit (ICU) who are frequently intubated, restricting mobility and potentially impeding the acquisition of high-quality echocardiographic images. In such cases, obtaining certain echocardiographic views may be challenging. Studies have demonstrated that a subjective and visual evaluation of ejection fraction, acquired through the parasternal long-axis view (PLAX), is satisfactory for critically ill patients. POCUS plays a supportive role in this visual assessment, offering a practical and effective means of evaluating cardiac function in ICU patients, where traditional echocardiography may be more logistically challenging [6,20].

Beyond visual assessment, the identification of left atrial enlargement (LAE) through POCUS serves as a significant clinical indicator of increased LV filling pressures that bear prognostic importance, particularly in patients with certain medical conditions, such as cardiomyopathy, mitral valve disease, and atrial fibrillation. POCUS facilitates a rapid assessment of the left atrial anteroposterior diameter, allowing for a quick comparison to a 4 cm reference standard using the PLAX [6,21]. Moreover, POCUS aids in the straightforward detection of pulmonary vascular congestion and pleural effusions, contributing to the identification of acute decompensated heart failure [22]. The ability to identify pleural effusions is an additional benefit of POCUS, providing comprehensive information about cardiac and pulmonary status in a timely manner [6,21]. This multifaceted capability of POCUS contributes to its effectiveness as a diagnostic tool in assessing and managing various cardiac conditions.

A study has reported that the use of POCUS in detecting LV dysfunction results in a reduction in the time taken to implement appropriate medical management compared to standard echocardiography. The average time difference reported in the study was 18 hours [6,11]. This finding underscores the timeliness and efficiency of POCUS in facilitating quicker diagnostic assessments, leading to prompt initiation of medical interventions. The ability of POCUS to provide rapid insights into cardiac function contributes to more expedited decision-making and treatment strategies, particularly in critical care and emergency settings.

Assessment of Valvular Conditions

Detecting valvular disease through physical examination is constrained by the examiner's proficiency, the patient's body composition, the severity of lesions, and the number of valvular lesions. In cases where there is uncertainty regarding the identification of a murmur, POCUS can support providing more information [6]. The POCUS can identify significant valvular stenosis by presenting limited motion of the aortic and mitral valve leaflets in captured images [6,21]. Color Doppler can be applied in POCUS to reveal turbulence across the affected valve, indicating potentially significant stenosis [6].

The ability to detect valvular disease through physical examination is constrained by factors, such as the skill of the examiner, the physique of the patient, the seriousness of lesions, and the presence of multiple valvular lesions. In cases where uncertainty arises in identifying a murmur, POCUS serves as a valuable tool, providing additional information [6]. POCUS can identify significant valvular stenosis by showcasing restricted motion of aortic and mitral valve leaflets in acquired images [21]. In addition, color Doppler imaging, a feature of POCUS, can be employed to visualize turbulence through the affected valve. This turbulent flow can be indicative of clinically significant stenosis [6].

POCUS proves valuable in the detection of regurgitant lesions, particularly through 2D visualization of valvular structures. Observing mitral valve prolapse or improper leaflet coaptation offers indications to the examiner regarding the existence of a regurgitant condition. This capability enhances the diagnostic utility of POCUS, allowing for a more detailed assessment of valvular function. Furthermore, POCUS can be effectively employed in the detection of valvular insufficiency in patients with endocarditis [6]. The real-time imaging provided by POCUS enables clinicians to identify and assess the extent of regurgitant lesions, contributing to the diagnostic process and aiding in the management of patients with valvular abnormalities, particularly in the context of infectious endocarditis.

Assessment of RV Function

Physical examination findings that suggest RV enlargement, such as a left parasternal heave and right-sided S3, are relatively infrequent and may have limited accuracy. In cases where there is suspicion of pulmonary embolism (PE) and a hypotensive patient is being evaluated, the assessment of right-sided cardiac structures using POCUS proves to be beneficial. Using POCUS, images of the right ventricle can be acquired through various views, including the PLAX, parasternal short-axis (PSAX), apical four-chamber, and subcostal views. This comprehensive evaluation aids in the assessment of RV size and function, providing valuable information in cases of suspected PE. In the ICU setting, the use of POCUS in evaluating a hypotensive patient is particularly helpful, particularly in cases where the patient may lack sufficient hemodynamic stability for a CT scan. POCUS allows for a rapid and bedside assessment, facilitating timely decision-making in critically ill patients suspected of having a pulmonary embolism [6].

Assessment of Pericardial Disease

Pericardial effusions can manifest with a diverse range of presentations, spanning from dyspnea to cardiogenic shock. Evaluating the existence of pericardial effusion or cardiac tamponade is especially critical in hypotensive patients. POCUS serves as a valuable tool for the rapid evaluation of pericardial effusion in both ICU and emergency department settings [6]. Research findings indicate a notable level of sensitivity and specificity in identifying effusions through POCUS, even when conducted by individuals without specialized training in cardiology. This highlights the effectiveness of POCUS in quickly and accurately identifying pericardial effusions, allowing for prompt intervention and management in patients with hypotension and suspected cardiac tamponade [6,23]. The ability to perform timely assessments at the bedside enhances the diagnostic capabilities of healthcare professionals, leading to more immediate and targeted patient care.

Assessment of Volume Status

In patients experiencing critical illness, evaluating hypovolemia and fluid responsiveness is of paramount importance. Clinical findings from physical examination, such as distention of jugular veins, demonstrate sensitivity and specificity but might have limitations in specific patient groups, including morbidly obese individuals with thick necks and intubated patients [21]. POCUS proves valuable in these situations by aiding in the assessment of the inferior vena cava (IVC), offering an accurate evaluation of intravascular volume status. Ultrasound can be utilized to measure the elevation of the jugular venous column when examining the neck veins physically proves to be difficult or inconclusive [6]. This application of POCUS enhances the ability to determine volume status, particularly in cases where traditional physical examination findings may be limited or difficult to interpret. The use of POCUS in assessing fluid responsiveness contributes to more precise and tailored management of critically ill patients.

Limitations of POCUS

POCUS has been instrumental in reporting substantial changes in medical management through assessments of various cardiac structures, including function of the left ventricle, function of the right ventricle, pericardial effusion and tamponade, fluid volume status, and abnormalities in heart valves. However, it is important to note that the accuracy of detecting these structural abnormalities through POCUS can vary. A summary of POCUS findings in structural abnormalities and their limitations is provided in Table 2.

**Table 2:** POCUS findings in structural abnormalities and its limitations AP: anteroposterior, CVP: central venous pressure, ECG: electrocardiogram, EPSS: E-point septal separation, IVC: inferior vena cava, LA: left artery, LV: left ventricular, PLAX: parasternal long-axis view, PSAX: parasternal short-axis view, RV: right ventricular Citation: Selby et al. [6]

Structural abnormality	Parameters	Typical view	Limitation
LV function	Visual estimation, EPSS	PLAX, apical	Poor windows, apical foreshortening
Left atrial enlargement	AP diameter, aorta-to-LA ratio	PLAX	Poor windows
V function	RV enlargement, hypokinesis, septal flattening	PSAX, apical	Apical foreshortening
Valvular disease	Leaflet restriction, leaflet coaptation, calcification, color Doppler findings	PLAX, apical	Absence of spectral Doppler capability on the majority of devices
Pericardial effusion	Visual assessment of the effusion size, RA/RV collapse	Subcostal	Incapability to measure effusion, absence of spectral Doppler, M-mode, and ECG gating preventing the determination of tamponade
CVP	IVC plethora, respiratory variation	Subcostal	Limited visibility or challenging imaging conditions in patients who are intubated

## Conclusions

Cardiac POCUS is a diagnostic modality to evaluate the heart and its blood vessels, performed by healthcare practitioners at the patient's bedside. At present, numerous medical specialists employ cardiac POCUS, and its utilization is expanding across various disciplines. Demonstrating its efficacy, cardiac POCUS functions as a valuable complement to the physical examination, especially in patients in critical condition. The ability of POCUS to provide early diagnoses has a substantial impact on medical management, contributing to a more timely initiation of appropriate interventions. As technology advances, cardiac POCUS is undergoing continuous improvements, with enhancements in image quality and functionality. These advancements are expected to further enhance the diagnostic capabilities of cardiac POCUS, making it an increasingly valuable tool in the hands of healthcare professionals across diverse medical specialties.
